# Acute Lung Injury and Acute Kidney Injury Are Established by Four Hours in Experimental Sepsis and Are Improved with Pre, but Not Post, Sepsis Administration of TNF-α Antibodies

**DOI:** 10.1371/journal.pone.0079037

**Published:** 2013-11-12

**Authors:** Rhea Bhargava, Christopher J. Altmann, Ana Andres-Hernando, Ryan G. Webb, Kayo Okamura, Yimu Yang, Sandor Falk, Eric P. Schmidt, Sarah Faubel

**Affiliations:** 1 Division of Renal Diseases and Hypertension, University of Colorado School of Medicine, Aurora, Colorado, United States of America; 2 Program in Translational Lung Research, Division of Pulmonary Sciences and Critical Care Medicine, Aurora, Colorado, United States of America; University of Colorado Denver, United States of America

## Abstract

**Introduction:**

Acute kidney injury (AKI) and acute lung injury (ALI) are serious complications of sepsis. AKI is often viewed as a late complication of sepsis. Notably, the onset of AKI relative to ALI is unclear as routine measures of kidney function (BUN and creatinine) are insensitive and increase late. In this study, we hypothesized that AKI and ALI would occur simultaneously due to a shared pathophysiology (i.e., TNF-α mediated systemic inflammatory response syndrome [SIRS]), but that sensitive markers of kidney function would be required to identify AKI.

**Methods:**

Sepsis was induced in adult male C57B/6 mice with 5 different one time doses of intraperitoneal (IP) endotoxin (LPS) (0.00001, 0.0001, 0.001, 0.01, or 0.25 mg) or cecal ligation and puncture (CLP). SIRS was assessed by serum proinflammatory cytokines (TNF-α, IL-1β, CXCL1, IL-6), ALI was assessed by lung inflammation (lung myeloperoxidase [MPO] activity), and AKI was assessed by serum creatinine, BUN, and glomerular filtration rate (GFR) (by FITC-labeled inulin clearance) at 4 hours. 20 µgs of TNF-α antibody (Ab) or vehicle were injected IP 2 hours before or 2 hours after IP LPS.

**Results:**

Serum cytokines increased with all 5 doses of LPS; AKI and ALI were detected within 4 hours of IP LPS or CLP, using sensitive markers of GFR and lung inflammation, respectively. Notably, creatinine did not increase with any dose; BUN increased with 0.01 and 0.25 mg. Remarkably, GFR was reduced 50% in the 0.001 mg LPS dose, demonstrating that dramatic loss of kidney function can occur in sepsis without a change in BUN or creatinine. Prophylactic TNF-α Ab reduced serum cytokines, lung MPO activity, and BUN; however, post-sepsis administration had no effect.

**Conclusions:**

ALI and AKI occur together early in the course of sepsis and TNF-α plays a role in the early pathogenesis of both.

## Introduction

Sepsis occurs in 650,000 to 750,000 patients in the United States annually [1], [2] and is the leading cause of death in non-coronary intensive care units (ICUs). Acute kidney injury (AKI) and acute lung injury (ALI) are particularly common complications of sepsis and the development of either increases mortality. Currently, there is growing interest in the potential cross talk that exists between injured organs, particularly in regard to the relationship between AKI and ALI [3], [4] with one organ causing or contributing to injury to another. Animal studies demonstrate that AKI can cause ALI [5], [6], [7], [8], and that ALI can cause AKI [9]. With regard to sepsis, however, little is known about the relationship between AKI and ALI. If fact, the development of both ALI and AKI has not been carefully examined in animal or human sepsis and the onset of AKI relative to ALI is unknown. Since the prognosis of critically ill patients with sepsis is based on organ failure assessment, a better understanding of the development of organ injury in sepsis is clinically needed [10]. A barrier to the study of AKI in sepsis is that routine measures of kidney function (BUN and creatinine) are insensitive [11] and increase late in the course of disease [12]. Therefore, AKI is typically recognized as a late complication of sepsis, often prompting withdrawal of care [13].

Although several mechanisms are involved in the development of ALI and AKI in sepsis, it is likely that several aspects of the pathophysiology are shared. For example, it has long been accepted that proinflammatory cytokines resulting in the systemic inflammatory response syndrome (SIRS) is a key event in the development of organ injury and death in patients with sepsis [14]. In particular, the proinflammatory cytokine TNF-α has been shown to mediate SIRS, ALI, and AKI [15], [16], [17] in animal models of sepsis. There is now controversy regarding the role of TNF-α and the SIRS response in the pathophysiology of organ injury after sepsis and the role of animal models used to study sepsis has been questioned. Central to the controversy is the apparent failure of clinical trials of anti-TNF-α therapies in sepsis, which were based on animal studies. In fact, the pendulum of the debate has now swung so far that is it being argued that, rather than mediating organ injury, the SIRS response in sepsis is “always good [18].”

In the present study, we sought to examine the onset of AKI relative to ALI in experimental sepsis. We hypothesized that AKI and ALI would occur simultaneously due to a shared pathophysiology (i.e., TNF-α mediated systemic inflammatory response syndrome [SIRS]), but that sensitive methods would be required to identify AKI. Indeed, we found that AKI and ALI were evident by 4 hours after sepsis. To investigate the role of TNF-α in the pathophysiology of both, we tested the effect of anti-TNF-α therapy administered before or after the onset of sepsis; we hypothesized that pretreatment with anti-TNF-α therapy would be effective, due to shared pathophysiology of injury, but that anti-TNF-α treatment initiated after the onset of sepsis would be ineffective due to rapidly established organ injury. As expected, prophylactic anti-TNF-α therapy improved kidney function and reduced ALI, however, post-sepsis administration had no effect on either parameter.

## Materials and Methods

### Animals

8- to 10-week-old, male C57BL/6 mice (Jackson Laboratories, Bar Harbor, ME) that weighed 20 to 25 grams were used. Mice were maintained on a standard diet, and water was freely available. All experiments were conducted with adherence to the National Institutes of Health Guide for the Care and Use of Laboratory Animals. The animal protocol was approved by the Animal Care and Use Committee of the University of Colorado, Denver.

### Sepsis Models

#### Intraperitoneal endotoxin administration

0.00001, 0.0001, 0.001, 0.01, or 0.25 mg of endotoxin (Sigma Aldrich, Escherichia coli 0111:B4 catalog number L2630) in 100 µL of sterile saline was injected once, intraperitoneally (IP). Vehicle was sterile saline.

#### Cecal ligation and puncture

Cecal ligation and puncture (CLP) and sham surgery were performed as previously described [19].

Mice were sacrificed at 4 or 24 hours post procedure for serum cytokines, lung myeloperoxidase (MPO) activity, urine neutrophil gelatinase-associated lipocalin (NGAL) and urine IL-6. Separate experiments were performed for glomerular filtration rate (GFR) measurements 4 and 24 hours post procedure.

### Collection and Preparation of Serum Samples

At sacrifice, blood was obtained via cardiac puncture and serum was collected as previously described [6].

### Serum Cytokine Measurement

Serum IL-6, CXCL1, TNF-α, IL-1β, and IL-10 were determined using a mouse proinflammatory 7-plex ultra-sensitive kit (MesoScale Discovery, Gaithersburg, MD, USA) per manufacturer’s instructions.

### Lung Neutrophil Assessment

#### Lung myeloperoxidase (MPO) activity

Lung MPO activity was determined on ¼ lung section as previously described [6], [7].

#### Flow cytometry for lung neutrophils

Lung parenchyma was minced and processed by enzymatic digestion: 2 mg/ml collagenase (Roche) in RPMI media (Gibco), incubated, strained, and RBCs were lysed with ACK red blood cell lysis buffer (Quality Biological), and washed. Cells were stained with: CD11c-PECy7, CD11b-Pacific blue, F4/80-PerCP-Cy5 (eBiosciences), CD45-V500, and Ly6G-APCCy7 (BD Biosciences) and fixed in 200 µl of 1% paraformaldehyde (Sigma). Neutrophils were identified as CD45, CD11b and Ly6G positive and F480 negative.

### Assessment of Kidney Function/kidney Injury

#### Serum creatinine and Blood Urea Nitrogen (BUN)

Serum creatinine and BUN were measured using a VetAce autoanalyzer (Alfa Wassermann, West Caldwell, NJ).

#### Urine Neutrophil Gelatinase-associated Lipocalin (NGAL) and urine IL-6

Urine NGAL and urine IL-6 were measured by ELISA (R&D Systems) following the manufacturer’s instructions.

#### Glomerular Filtration Rate (GFR) measurement

GFR was measured by inulin clearance, as previously described [20]. Briefly, mice were anesthetized with IP pentobarbital (60 µg/g body weight) and a jugular central venous catheter (PE-10) and bladder catheter were placed. 0.75% FITC-inulin in 2.25% BSA (in saline) was infused and two 30-min collections of urine and blood were obtained. FITC in plasma and urine samples was measured using a CytoFluor plate reader (BioTek Instruments, Winooski VT). GFR was defined as [inulin_urine_]*(urine output per 30 min)/[inulin_plasma_].

### TNF-α Antibody Administration

20 µgs of mouse anti-TNF-α monoclonal antibody (Clone MP6-XT22, R&D Systems, catalog number MAB4101) or IgG control (Clone 43414, R&D Systems, catalog number MAB005) in 100 µL of sterile PBS was injected IP into in the lower left abdominal quadrant either 2 hours before IP endotoxin injection or 2 hours after IP endotoxin injection.

### Statistical Analysis

All values are expressed as mean ± standard error (SE). Non-parametric t-tests were performed for the following experimental groups: vehicle injection versus IP endotoxin at the same time point, or sham surgery versus CLP, or vehicle treatment versus TNF-α antibody. Groups with significant variance were subjected to Welch’s correction. A *P* value of ≤0.05 was considered statistically significant.

## Results

### Serum Proinflammatory Cytokines after Intraperitoneal Endotoxin

Since we hypothesized that both AKI and ALI would occur at a similar time point after the onset of sepsis due to TNF-α-associated SIRS response, we sought to determine the onset of this proinflammatory response. Furthermore, we sought to determine the dose dependent effects of serum proinflammatory cytokine accumulation after IP endotoxin injection. Therefore, serum IL-6, CXCL1, TNF-α, and IL-1β were determined 4 and 24 hours after IP injection of vehicle (saline), and 5 different doses of endotoxin: 0.00001, 0.0001, 0.001, 0.01, and 0.25 mg (doses 1, 2, 3, 4, and 5 respectively).

**Figure 1:**
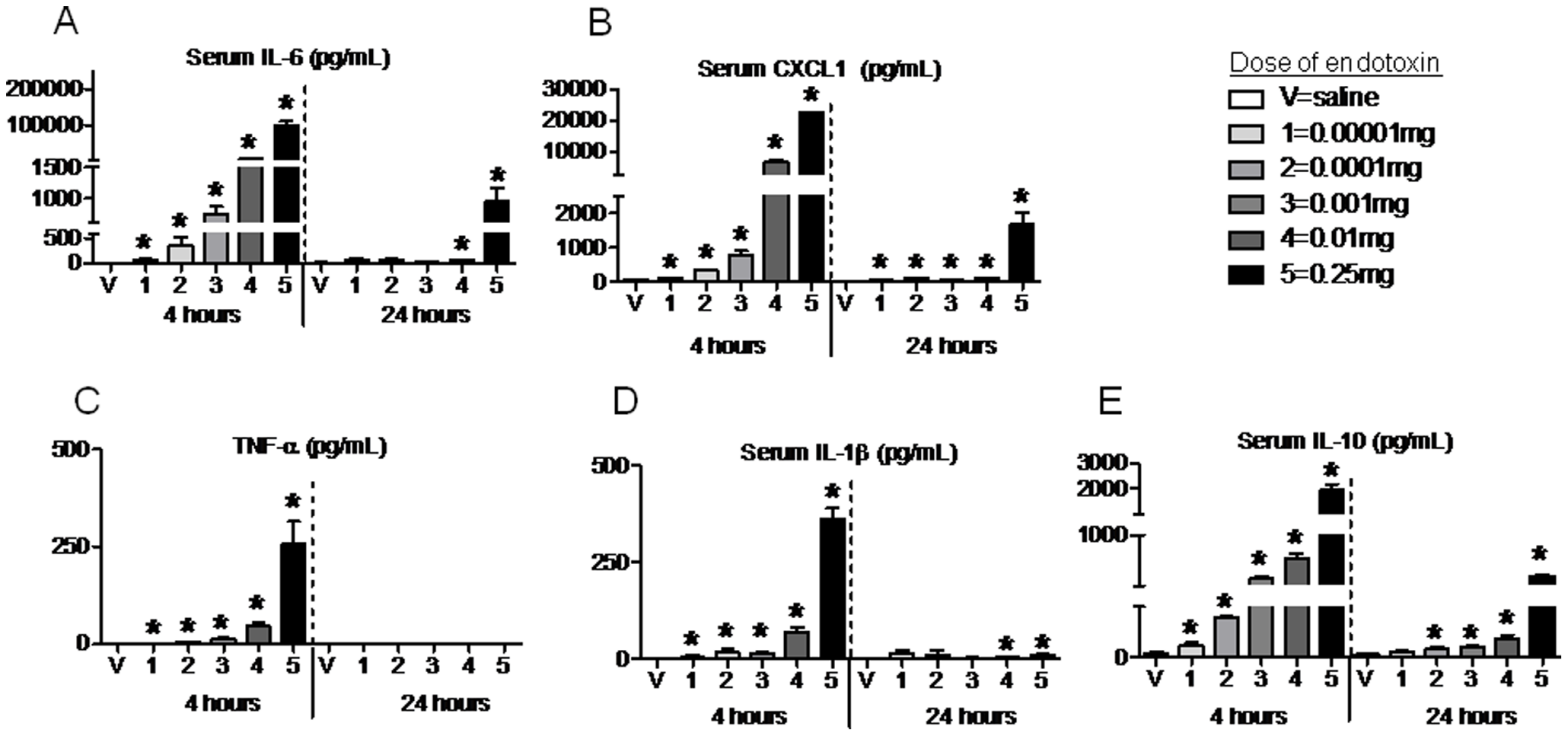
Serum cytokines 4 and 24 hours after 5 different doses of intraperitoneal endotoxin. Serum (A) IL-6, (B) CXCL-1, (C) TNF-α, (D) IL-1β, and (E) IL-10 were measured 4 and 24 hours after intraperitoneal saline injection (V, vehicle) or intraperitoneal injection of 5 different doses of endotoxin: 1 (0.00001 mg), 2 (0.0001 mg), 3 (0.001 mg), 4 (0.01 mg), and 5 (0.25 mg); n = 4–14. *P<0.05 versus V (saline) at the same time point.

As shown in Figure 1, the lowest dose of endotoxin resulted in a significant increase in all serum cytokines measured and serum cytokine levels increased with dose in a step-wise fashion.

### Serum IL-10 after Intraperitoneal Endotoxin

IL-10 is the prototypical anti-inflammatory cytokine which is representative of the initiation of the compensatory anti-inflammatory response (CARS). As shown in Figure 1E, serum IL-10 increased in a similar magnitude and fashion as proinflammatory cytokines after endotoxin administration. The CARS response is typically described as occurring “later” in the course of sepsis, however, these data suggest that SIRS and CARS in inflammatory insults occur nearly simultaneously. These results are similar to what we have observed after AKI alone, with upregulation of IL-10 production occurring within just two hours [21].

### Lung MPO Activity after Intraperitoneal Endotoxin

**Figure 2:**
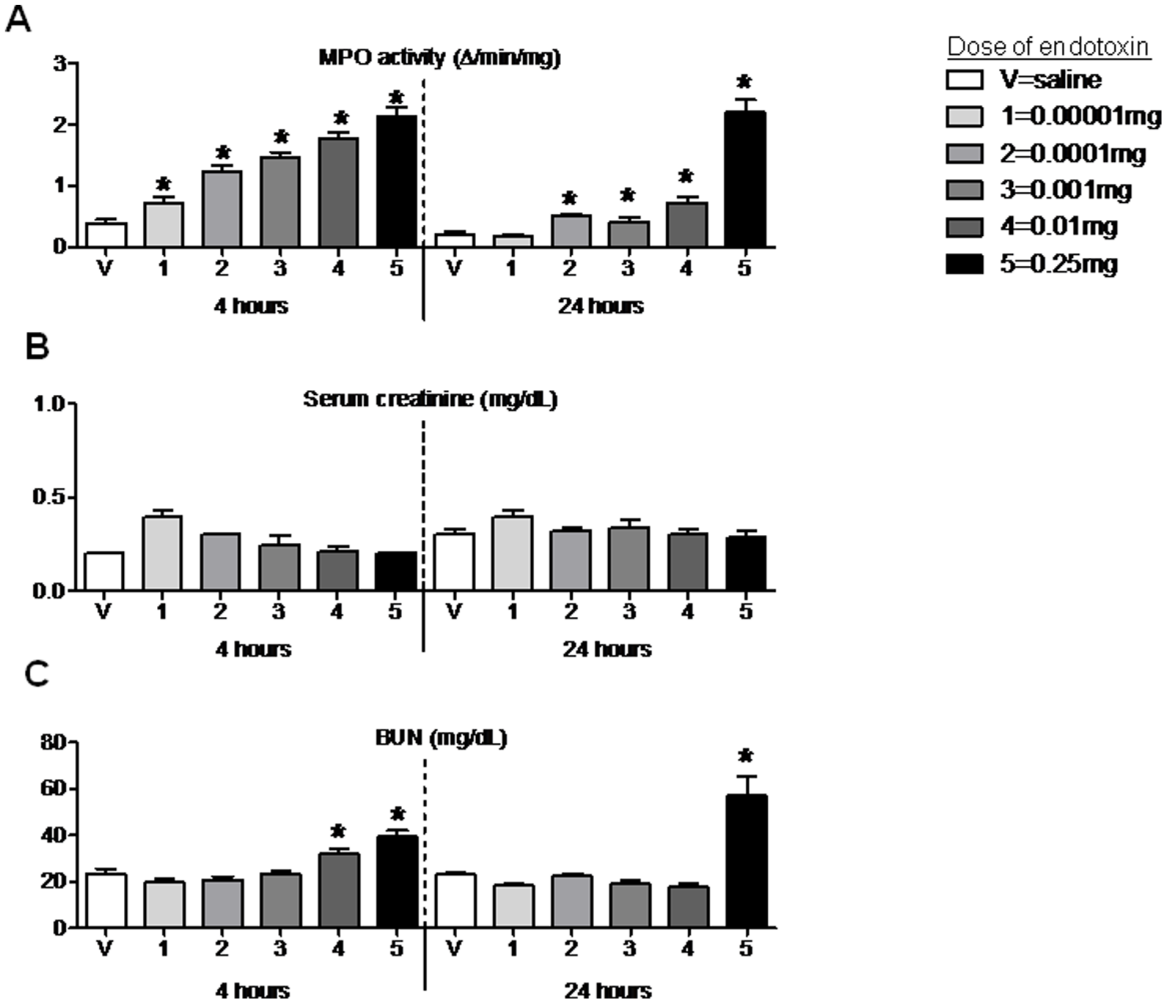
Lung MPO activity, serum creatinine, and BUN 4 hours after 5 different doses of intraperitoneal endotoxin. (A) Lung MPO activity, (B) serum creatinine, and (C) BUN were measured 4 and 24 hours (hr) after intraperitoneal saline injection (V, vehicle) or intraperitoneal injection of 5 different doses of endotoxin: 1 (0.00001 mg), 2 (0.0001 mg), 3 (0.001 mg), 4 (0.01 mg), and 5 (0.25 mg); n = 4–14. *P<0.05 versus V (saline) at the same time point.

As shown in Figure 2A, the increase in serum proinflammatory cytokines was parallel by a dose dependent increase in lung MPO activity. Lung MPO activity is a very sensitive marker of neutrophil accumulation in the lungs and probably reflects both neutrophil accumulation and neutrophil activation. Remarkably, even the very lowest dose of endotoxin resulted in an increase in lung MPO activity.

We wished to correlate the lung MPO activity with lung neutrophil accumulation. Therefore, for two of the endotoxin doses, 0.001 mg and 0.01 mg (doses 3, and 4 respectively) we confirmed the increase in MPO activity by flow cytometry for neutrophils; lung neutrophils were 9±1% in vehicle-treated, 28±2% in 0.001 mg treated (P<0.001 versus vehicle-treated), and 35±1% in 0.01 mg treated (P<0.001 versus vehicle-treated and <0.04 versus 0.001 mg treated). Thus, lung MPO activity is an accurate reflection of lung neutrophil accumulation.

We recognize that MPO activity and percent neutrophil accumulation represent acute lung inflammation, but not necessarily acute lung injury per se. Indeed, evidence suggests that factors in addition to lung neutrophil accumulation are necessary for lung injury to occur [22]. However, since lung neutrophil accumulation leading to subsequent tissue destruction is considered a key *initiation* factor in the development of acute lung injury and adult respiratory distress syndrome [23], we chose this endpoint to characterize the development of lung injury post endotoxin. In fact, lung MPO activity is regarded as one of the most sensitive markers of lung inflammation available, thus we believe lung MPO activity is a suitable marker of lung inflammation and, at the very least, the *initiation* of lung injury post endotoxin injection and post CLP.

### Serum Creatinine and BUN after Intraperitoneal Endotoxin

To assess renal dysfunction post endotoxin injection, we first examined serum creatinine and BUN (Figure 2B,C). Although serum cytokines and lung inflammation were increased with the lowest dose of endotoxin tested, no change in creatinine was observed at any dose post endotoxin injection at either 4 or 24 hours; BUN was modestly increased 4 hours post-injection after the two highest doses tested (i.e., 0.01 mg and 0.25 mg); BUN was persistently increased at 24 hours in only the highest dose of endotoxin tested (i.e., 0.25 mg).

### Glomerular Filtration Rate after Intraperitoneal Endotoxin

**Figure 3:**
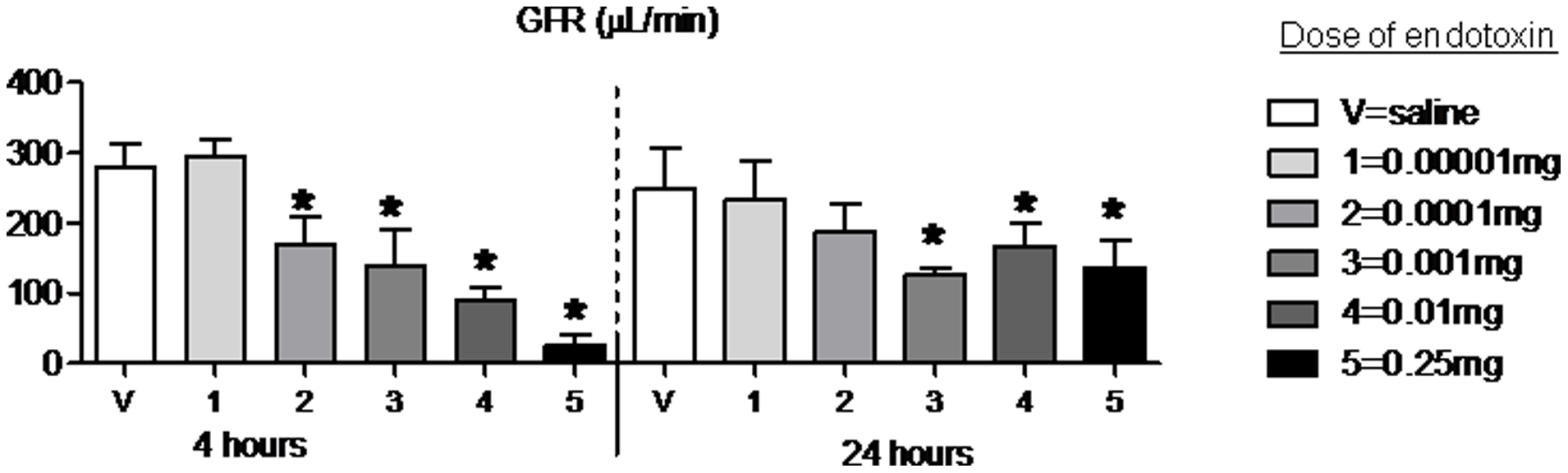
Glomerular filtration rate (GFR) 4 and 24 hours after 5 different doses of intraperitoneal endotoxin. GFR, by FITC-labeled inulin clearance, was determined 4 and 24 hours (hr) after intraperitoneal saline injection (V, vehicle] or intraperitoneal injection of 5 different doses of endotoxin: 1 (0.00001 mg), 2 (0.0001 mg), 3 (0.001 mg), 4 (0.01 mg), and 5 (0.25 mg); n = 4–14. *P<0.05 versus V (saline) at the same time point.

Since creatinine is well known to be a poor marker of kidney function in sepsis, we questioned whether significant decreases in renal function may occur below the detection rate of both BUN and creatinine. Therefore, we measured the gold standard of renal function, GFR, after IP endotoxin injection. Although no change in GFR was noted with the lowest dose of endotoxin tested, doses 2, 3, 4 and 5 all caused in a significant decrease in GFR compared to vehicle-injected (i.e., baseline) as shown in Figure 3.

Notably, the middle dose of endotoxin (i.e., dose 3–0.001 mg) was associated with a GFR that was 50% reduced compared to vehicle-injected even though neither serum creatinine nor BUN increased with this dose; specifically, GFR (µL/min) was 280.7±33.9 in vehicle-injected versus 140.4±51.4 in 0.001 mg (i.e., dose 3) endotoxin injected, P = 0.04.

GFR was persistently decreased at 24 hours for doses 3, 4 and 5 (i.e., 0.001, 0.01, and 0.25 mg, respectively).

### Urine NGAL after Intraperitoneal Endotoxin

**Figure 4:**
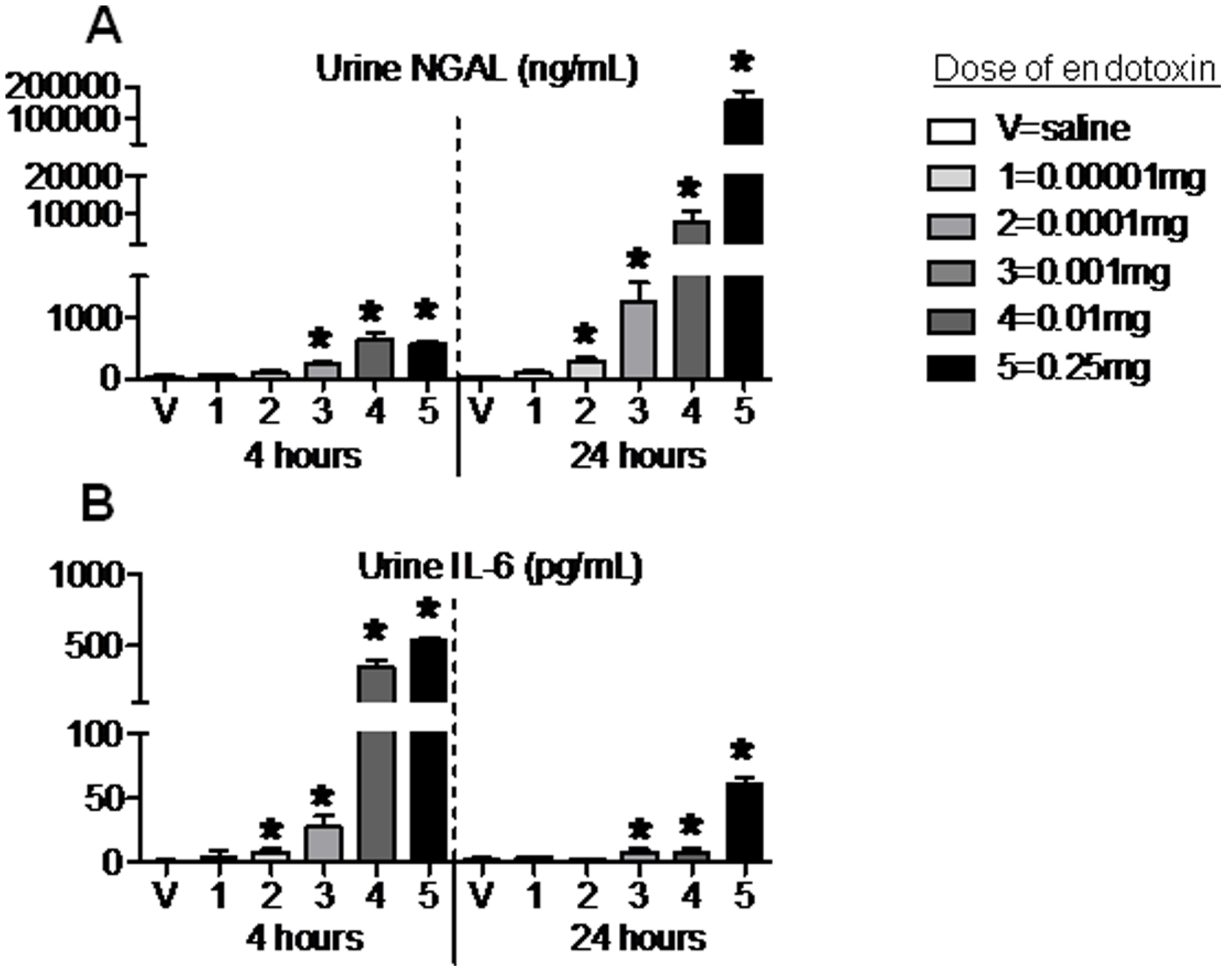
Urine neutrophil gelatinase-associated lipocalin (NGAL) and urine IL-6 4 and 24 hours after 5 different doses of intraperitoneal endotoxin. (A) Urine NGAL, and (B) urine IL-6 were measured 4 and 24 hours (hr) after intraperitoneal saline injection (V, vehicle) or intraperitoneal injection of 5 different doses of endotoxin: 1 (0.00001 mg), 2 (0.0001 mg), 3 (0.001 mg), 4 (0.01 mg), and 5 (0.25 mg); n = 4–14. *P<0.05 versus V (saline) at the same time point.

**Figure 5:**
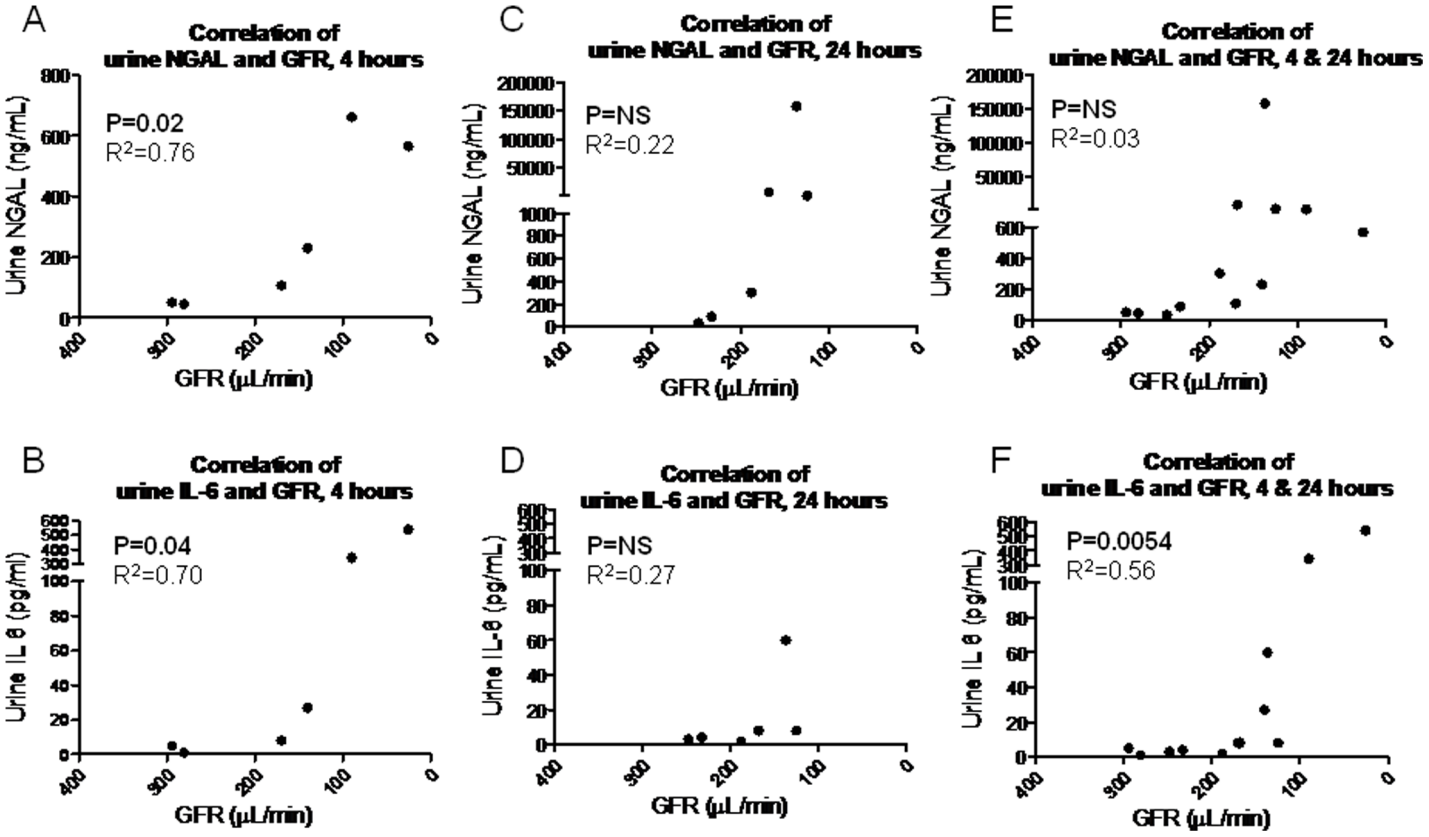
Performance of urine NGAL and urine IL-6 to reflect changes in glomerular filtration rate (GFR) after intraperitoneal endotoxin injection. Mean GFR were correlated with mean urine NGAL and mean urine IL-6 values at 4 hours only, 24 hours only, and combined values for 4 and 24 hours. (A) Urine IL-6, and (B) urine NGAL correlated well with GFR at 4 hours post endotoxin injection. (C) Urine NGAL and (D) urine IL-6 did not correlate with GFR at 24 hours post endotoxin injection. (E) Urine NGAL and (F) urine IL-6 performed poorly overall as markers of GFR when the 4 and 24 hours values were combined.

Because of the poor performance of serum creatinine and BUN to detect the fall in GFR post-endotoxin injection, we examined whether urine NGAL might be a more sensitive marker of renal dysfunction post-endotoxin injection. As shown in Figure 4, at the 4 hour time point, urine NGAL levels increased beginning with dose 3 and persisting with doses 4 and 5 despite no increase in creatinine and an increase in BUN only for doses 4 and 5. Additionally, mean GFRs and mean urine NGAL values were significantly correlated with a P value of 0.02 and an excellent R^2^ of 0.76 (Figure 5).

At the 24 hour time point, urine NGAL increased in a dose dependant fashion relative to the dose of endotoxin (Figure 4), but showed no correlation with GFR (P = NS, R^2^ = 0.22) (Figure 5). Thus, the overall correlation between urine NGAL and GFR (both the 4 and 24 hour time points) was not significant (P = NS) with poor correlation (R^2^ = 0.03).

### Urine IL-6 after Intraperitoneal Endotoxin

We have previously shown that urine IL-6 increases early in mice and patients with ischemic AKI [24]. Therefore, we questioned whether urine IL-6 might also be a marker of the fall in GFR post-endotoxin injection. Urine IL-6 performed well as a sensitive marker of kidney function at the 4 hour time point with an increase beginning with the 2^nd^ dose of endotoxin (Figure 4). Mean GFRs and mean urine IL-6 values were significantly correlated with a P value of 0.04 and an R^2^ of 0.70 (Figure 5). At the 24 hour time point, however, the correlation was weaker with an R^2^ of 0.17; overall (4 and 24 hour values), the correlation between urine IL-6 and GFR was significant with a P value of 0.0064 and an R^2^ of 0.56, however, this significance appears to be driven by the results at the 4 hour time point (Figure 5).

### Urine IL-6 after IV Injection of Recombinant Murine IL-6 to IL-6 Deficient (−/−) Mice after Endotoxin Injection

In patients, increased serum IL-6 predicts AKI in both ischemic AKI [25] as well as sepsis-induced AKI [26]. We have previously demonstrated that a source of urine IL-6 in ischemic AKI is the circulation. Utilizing IV injection of recombinant IL-6 in ischemic AKI, we found that circulating IL-6 appeared in the urine in ischemic AKI that is probably due to filtration and subsequent failure of proximal tubule metabolism due to proximal tubular injury [24]. To determine if circulating IL-6 appeared in the urine in sepsis-induced AKI for the same reasons, we performed a similar experiment. Specifically, 24 hours after IP injection of 0.25 mg of endotoxin to IL-6 deficient mice, we administered 200 ng of recombinant murine IL-6 (Peprotech) intravenously; mice were placed in metabolic cages and urine was collected for 1 hour after IV IL-6 injection. Unlike with ischemic AKI, IL-6 was *not* detected in the urine which demonstrates that filtration and subsequent failure of proximal tubule metabolism was not responsible for the increase in urine IL-6 in sepsis; these data also suggest that proximal tubule function may be more intact in sepsis-induced AKI versus ischemic AKI.

### Serum Proinflammatory Cytokines after Cecal Ligation and Puncture (CLP)

A criticism of the IP endotoxin experimental model is that it may not mimic the complexity of true bacterial sepsis. Therefore, to further explore the role of proinflammatory cytokines in the development of AKI and ALI in sepsis, we studied the more robust sepsis model of cecal ligation and puncture.

**Figure 6:**
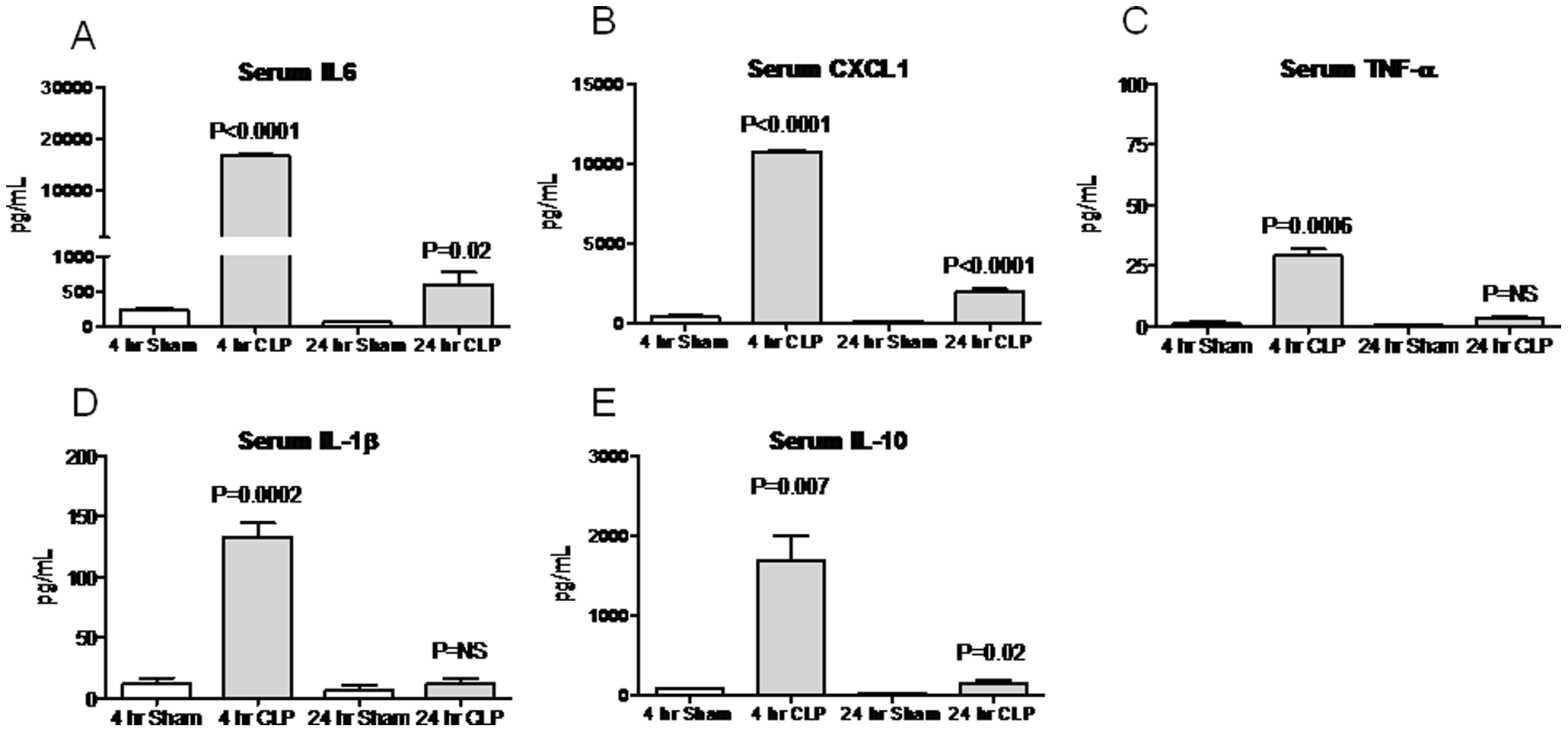
Serum cytokines 4 and 24 hours after cecal ligation and puncture (CLP). Serum (A) IL-6, (B) CXCL-1, (C) TNF-α, (D) IL-1β, and (E) IL-10 were measured 4 and 24 hours (hr) after sham operation (Sham) and CLP; n = 5–10. P values are versus Sham at the same time point.

As shown in Figure 6, the serum proinflammatory cytokines IL-6, CXCL1, TNF-α, and IL-1β increased dramatically by 4 hours after CLP and were significantly reduced 24 hours after CLP, a pattern similar to IP endotoxin administration. In fact, the cytokine levels were very similar to dose 4 of IP endotoxin (i.e, 0.01 mg).

### Serum IL-10 after Cecal Ligation and Puncture (CLP)

As with IP endotoxin administration, CLP resulted in a dramatic increase in serum IL-10 4 hours post procedure and the serum level was decreased by 24 hours Figure 6). As with proinflammatory cytokines, serum IL-10 level was similar to dose 4 of IP endotoxin (i.e., 0.01 mg).

### Lung MPO Activity, Serum Creatinine, BUN, and GFR after Cecal Ligation and Puncture (CLP)

**Figure 7:**
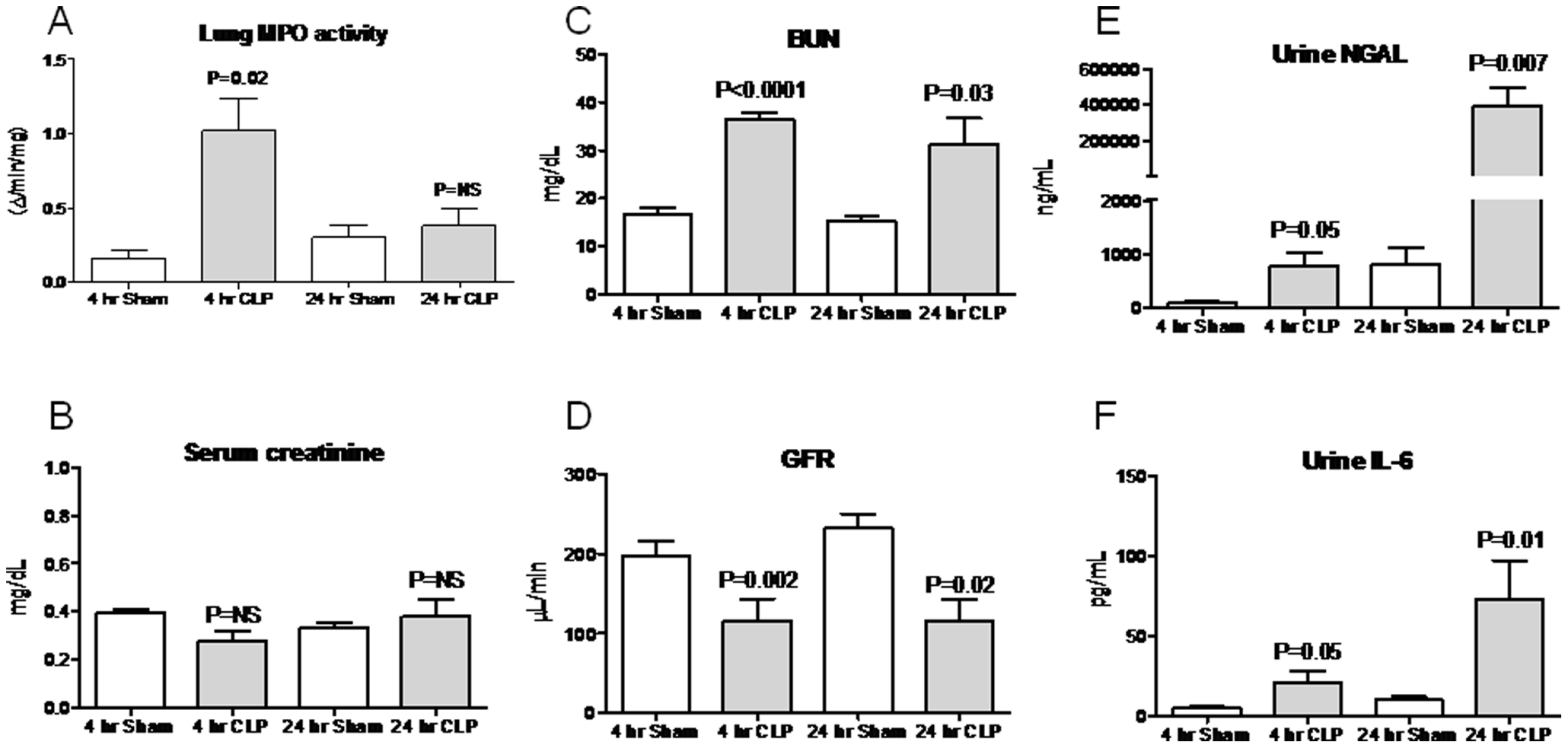
Lung MPO activity, serum creatinine, BUN, glomerular filtration rate (GFR), urine neutrophil gelatinase-associated lipocalin (NGAL), and urine IL-6 BUN 4 and 24 hours after cecal ligation and puncture (CLP). (A) Lung MPO activity, (B) Serum creatinine, (C) BUN, (D) GFR, (E) urine NGAL, and (F) urine IL-6 were determined 4 and 24 hours (hr) after sham operation (Sham) and CLP; n = 5–10. P values are versus Sham at the same time point.

As with IP endotoxin, CLP resulted in an increase in lung MPO activity and BUN by 4 hours post procedure and the increase in BUN correlated with a decrease in GFR (Figure 7). As with the serum cytokines at 4 hours, these data regarding MPO activity, BUN, and GFR are most similar to dose 4 of IP endotoxin. At 24 hours, lung MPO activity was near baseline, although kidney function was still reduced as judged by both BUN and GFR (Figure 7).

### Urine NGAL and Urine IL-6 after Cecal Ligation and Puncture (CLP)

As with IP endotoxin, urine NGAL was increased at the 4 hour time point, and dramatically increased at 24 hours post-CLP (Figure 7). Similarly, urine IL-6 was increased at 4 and 24 hours. Thus, like the IP endotoxin model, urine NGAL and IL-6 seem to be more related to the course of sepsis than to the course of renal function during sepsis.

### Anti-TNF-α Administration 2 Hours before or 2 hours after the Onset of Experimental Sepsis with IP Endotoxin

**Figure 8:**
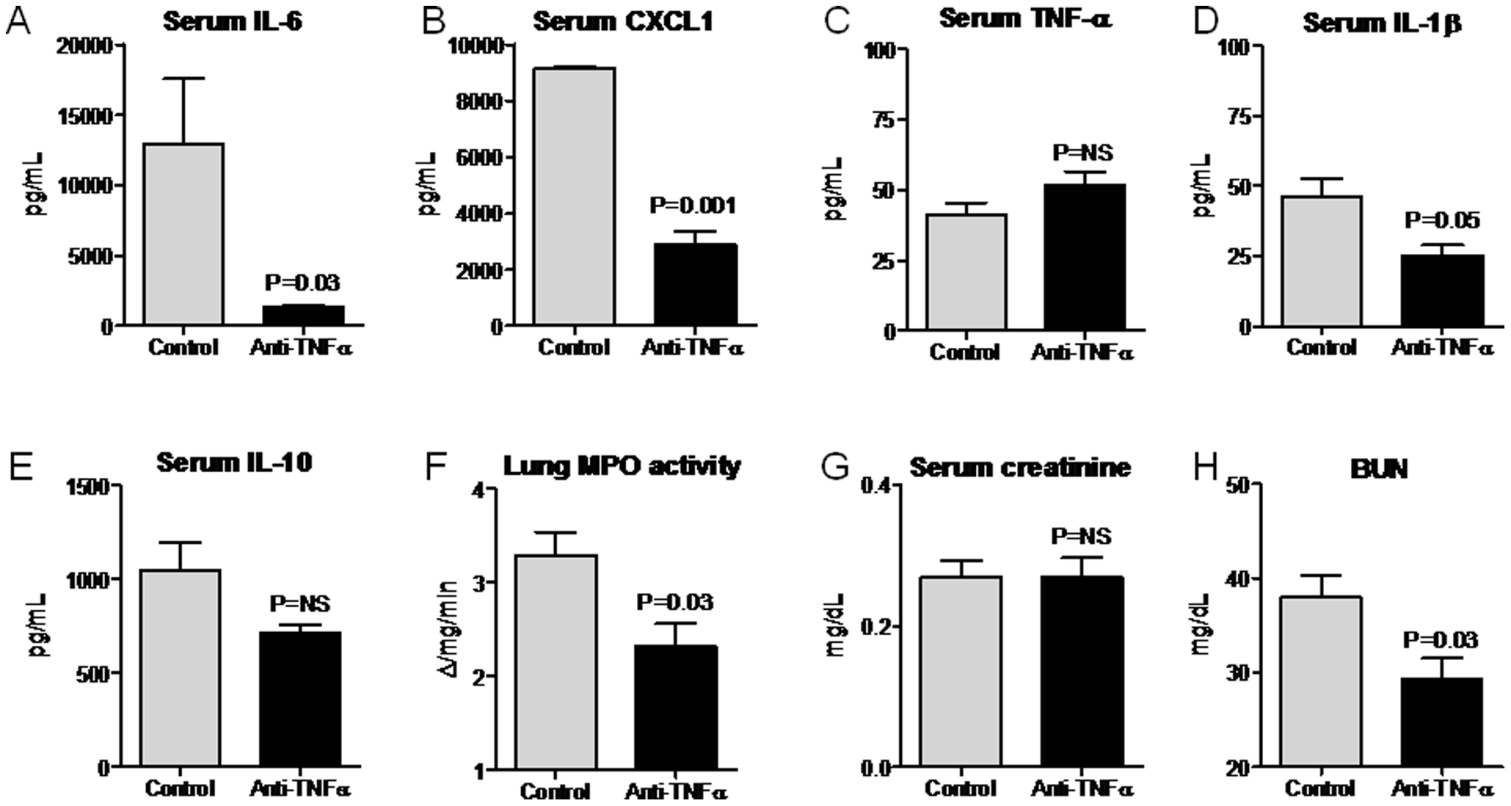
Serum cytokines, lung MPO activity, serum creatinine, and BUN in mice treated with anti-TNF-α therapy 2 hours before intraperitoneal (IP) endotoxin administration. Serum (A) IL-6, (B) CXCL1, (C) TNF-α, (D) IL-1β, (E) IL-10, and (F) lung MPO activity, and (G) serum creatinine, and (H) BUN were determined 4 hours after IP endotoxin in mice treated with anti-TNF-α antibody (Anti-TNF-α) or control 2 hours before endotoxin administration; n = 4–5. The serum proinflammatory cytokines IL-6, CXCL1, and IL-1β, lung MPO activity and BUN were all reduced by pre-treatment with anti-TNF-α antibody.

To determine whether the development of lung inflammation and AKI were related to the TNF-α mediated SIRS response, we administered TNF-α antibodies 2 hours prior to the administration of dose 4 of endotoxin (i.e., 0.01 mg). As shown in Figure 8, serum proinflammatory cytokines (IL-6, CXCL1, IL-1β), serum IL-10, lung MPO activity and BUN were all significantly reduced with TNF-α antibodies. These data demonstrate that TNF-α plays a key role in proinflammatory cytokine production, lung inflammation and kidney function early in the course of sepsis.

Interestingly, serum TNF-α was not reduced by anti-TNF-α antibody treatment; we surmise that this is due to the detection of TNF-α bound to antibody which has not yet been cleared but has rendered the TNF-α functionally inactive as judged by the marked reduction in all other markers of inflammation.

**Figure 9:**
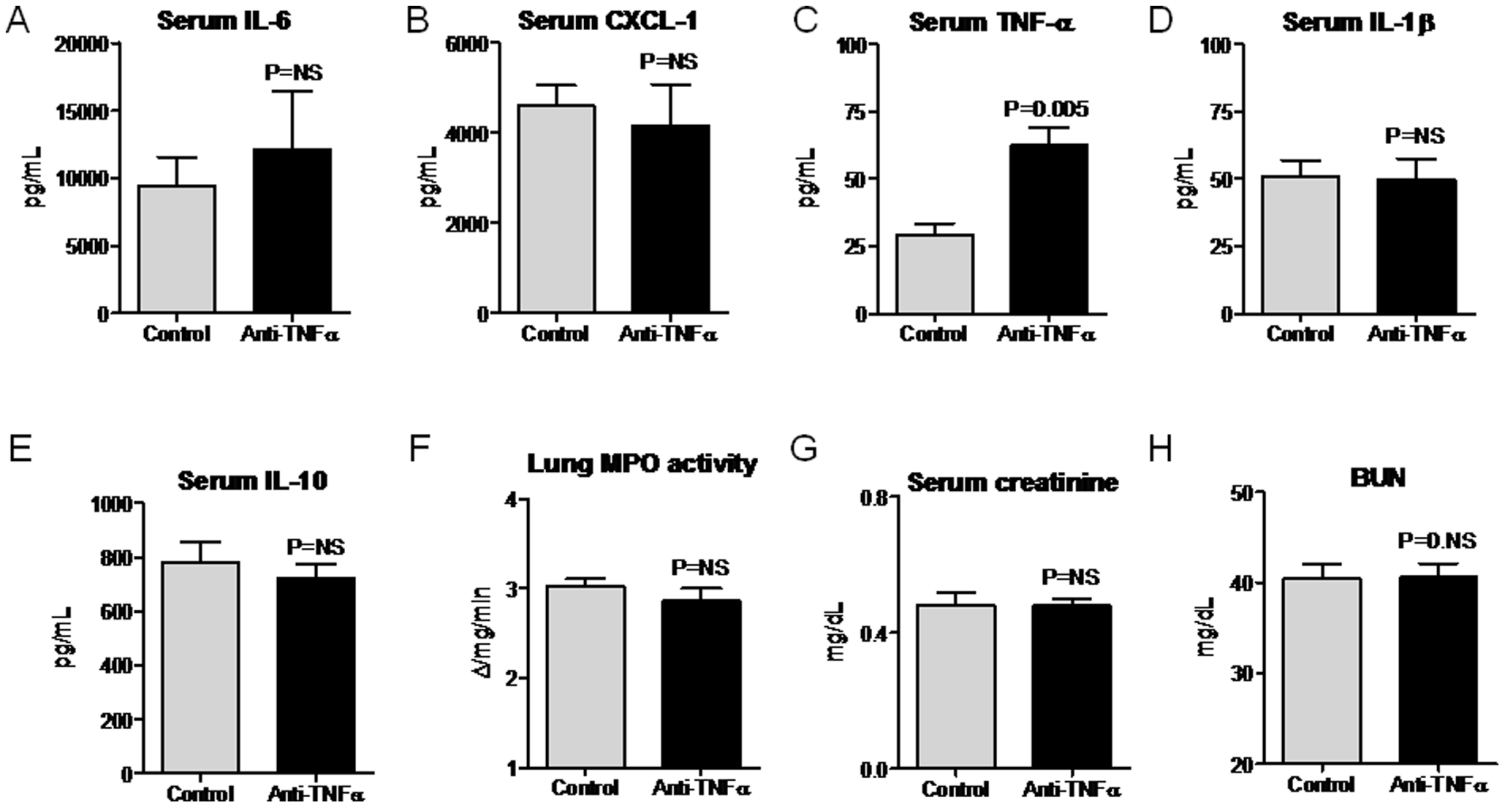
Serum cytokines, lung MPO activity, serum creatinine, and BUN in mice treated with anti-TNF-α therapy 2 hours after intraperitoneal (IP) endotoxin administration. Serum (A) IL-6, (B) CXCL1, (C) TNF-α, (D) IL-1β, (E) IL-10, and (F) lung MPO activity, and (G) serum creatinine, and (H) BUN were determined 4 hours after IP endotoxin in mice treated with anti-TNF-α antibody (Anti-TNF-α) or control 2 hours after endotoxin administration; n = 5. The serum proinflammatory cytokines IL-6, CXCL1, and IL-1β, serum IL-10, lung MPO activity, creatinine, and BUN were not affected by post-treatment with anti-TNF-α antibody.

Since lung inflammation and a decrement in kidney function were present by 4 hours post-endotoxin injection, we questioned whether TNF-α-mediated diseases might already be established, even at this early time point, and not amenable to anti-TNF-α therapy. To test this hypothesis, we administered anti-TNF-α antibodies just 2 hours after IP endotoxin injection. As shown in Figure 9, serum proinflammatory cytokines (IL-6, CXCL1, IL-1β), serum IL-10, lung MPO activity, and BUN were all not affected by anti-TNF-α therapy administered just 2 hours after IP endotoxin injection. Thus, TNF-α-mediated lung inflammation and kidney dysfunction are established early in the course of experimental sepsis.

## Discussion

In this report, we examined 5 different, one-time doses of IP endotoxin as well as cecal ligation and puncture (CLP) in order to characterize the onset and development of the systemic inflammatory response syndrome (SIRS), acute lung injury (ALI), and acute kidney injury (AKI) after sepsis; doses of endotoxin utilized were 0.00001, 0.0001, 0.001, 0.01, and 0.25 mg. For reference, a “low dose” endotoxin to study sepsis-induced AKI is 0.02 mg [27], [28]; moderate doses are 0.1 to 0.2 mg [16], [17], [29], and 0.25 mg is a typical dose [15], [30]. To study sepsis-induced ALI, doses of endotoxin fall into a similar range from 0.02 mg [19] to 0.25 mg [31]. In our study, SIRS was identified by serum proinflammatory cytokines, ALI was identified by lung inflammation, and AKI was identified by glomerular filtration rate (GFR). We found that just 0.0001 mg of IP endotoxin was sufficient to significantly increase serum proinflammatory cytokines, produce lung inflammation, and cause AKI. Although the onset of AKI relative to ALI in the course of sepsis was previously unclear, our data demonstrate that AKI and ALI occur together, rapidly at the onset of sepsis induced by either endotoxin injection or CLP. Our data lend significant insight into the development of AKI and ALI in sepsis, and the animal models used to study these complications.

Although it has been suggested that cytokine levels and the timing of appearance between human and experimental sepsis are different, our data suggest that this is not the case. For example, in one study, serum IL-6 levels at presentation were 796 pg/mL (range: 165 to 83,743) in non-survivors [32] of sepsis. In our study, mean serum IL-6 at four hours was: 72, 365, 732, 6210, and 98,923 pg/mL in the 0.00001, 0.0001, 0.001, 0.01, and 0.25 mg doses, respectively. Thus, the middle dose of endotoxin, 0.001 mg, resulted in cytokine levels similar to non-survivors of sepsis and was associated with significant lung inflammation and a 50% reduction in GFR. Even the high and low ranges identified at 4 hours in mice are not dissimilar to the high and low ranges seen in humans at the presentation of sepsis. Since higher levels of serum cytokines are associated with increased mortality in patients [32], our data in mice are consistent with the notion that higher serum cytokine levels promote worse organ injury.

Our data highlight the difficulty of the study of sepsis-induced AKI due to a failure of traditional (BUN, serum creatinine) and emerging biomarkers (urine IL-6, urine NGAL), to accurately reflect the severity of kidney function decline. Serum creatinine is the most widely used marker of AKI and modern definitions of AKI rely on an increase of serum creatinine to identify AKI [33]. Unfortunately, serum creatinine is well known to be a late marker of AKI in patients and Additionally, its performance in sepsis is especially poor due to reduced production of creatinine [11]; our data highlight this point as serum creatinine did not increase with even the most severe sepsis model (0.25 mg endotoxin) in which the GFR fell to 25 µL/min which is approximately 10% of normal. In general, severe disease is necessary for creatinine to increase in mouse models of kidney diseases because 30 to 60% of creatinine excretion in mice is due to secretion which further explains the lack of creatinine increase despite a severe decline in GFR in our study [34], [35]. Although we found that BUN did increase in the more severe models of sepsis, greater than a 50% loss of kidney function was required. These data suggest that in many cases of sepsis, significant kidney dysfunction may be present that is not identified because the creatinine does not increase. Although the clinical significance of loss of kidney function without an increase in creatinine in sepsis is not known, the weight of evidence suggests that small decrements of kidney function in AKI, in general, increase mortality and are associated with significant morbidity. Indeed, it has been shown that biomarker positive AKI, without an increase in creatinine, is also associated with adverse outcomes [36]. Thus, early recognition of AKI and an accurate way to measure kidney dysfunction in sepsis is urgently needed.

We examined two potential to biomarkers to identify AKI: urine IL-6 and urine NGAL, Urine IL-6 and urine NGAL both increased in sepsis-induced AKI; however, the levels did not correlate well with GFR. We have previously shown that urine IL-6 increases in patients and mice with AKI, in part, due to proximal tubular injury and impaired IL-6 metabolism [24]. Specifically, urine IL-6 correlated with GFR at 4, but not 24 hours; additionally, there was no evidence of impaired proximal tubule metabolism of IL-6. Urine NGAL, a well studied marker of AKI that reflects severity of tubular injury, also correlated with GFR at 4, but not 24 hours; in fact, even with recovery of GFR, urine NGAL was dramatically increased at 24 hours. Thus, urine NGAL appears to be a marker of the duration and severity of sepsis, rather than a specific marker of GFR; since NGAL is known to increase in inflammatory conditions, in general, the increase in urine NGAL post-LPS and post-CLP likely represents inflammation stimulated production in addition to AKI stimulated production [37]. Our data suggest that tubular injury, per se, may be less severe in sepsis-induced AKI than in other forms of AKI such as ischemia and that markers of tubular injury in general may not perform well in sepsis-induced AKI; indeed, measurement of real time GFR may hold the most promise for identifying AKI in patients with sepsis [38].

The development of AKI during the course of sepsis is known to increase mortality, although the reason for this association is unknown. Our data demonstrate that severe kidney dysfunction needs to be present to be detected with serum markers and that this was associated with the most severe models of experimental sepsis. Although AKI is known to have significant deleterious systemic effects [39], our data suggest that detection of AKI in sepsis may also be a reflection of the severity of sepsis which may explain the increased mortality risk associated with it. Along these lines, there is growing interest in the potential cross talk that may exist between AKI and ALI, with one form of injury contributing to the other. Since AKI is generally detected late in its course, it has been thought that ALI may contribute to AKI and experimental data supports this notion [9]. In the present study, we demonstrate that AKI and ALI are initiated together, due to a shared pathophysiology. Only at the very lowest dose of endotoxin was lung inflammation present without a decline in GFR, and this was mild; thus, we suggest that, at the onset of sepsis, if ALI is present then a reduction in GFR is also likely to be present, even if the BUN or creatinine are not increased.

The rapidity in which organ injury may become established after an insult has only recently been appreciated. For example, in the field of AKI, numerous therapies that showed promise in animal models failed in clinical trials [40]. However, it is now well understood that the clinical trials in patients were conducted in established disease [40]. Likewise, in the present study, we demonstrate that lung inflammation and AKI are well established by 4 hours post-endotoxin injection and post-CLP. In addition, our data show that the effect of TNF-α on lung inflammation and AKI occurs rapidly as prophylactic anti-TNF-α treatment was effective to reduce lung inflammation and kidney dysfunction, but anti-TNF-α treatment administered just two hours after the onset of sepsis was ineffective. The failure of anti-TNF-α [41] therapies to improve mortality in clinical trials of human sepsis is frequently cited as a failure of the animal model to replicate human sepsis; however, our data demonstrate that treatment of established disease, as was employed in clinical trials of sepsis, could not have been expected to be beneficial. Although the debate regarding the use of animal models in the study of sepsis will continue, we believe the failure of anti-TNF-α treatments in clinical trials cannot continue to be used as an argument supporting the failure of animal studies to replicate human sepsis.

## Conclusions

Our data demonstrate that the IP endotoxin model and CLP model of sepsis are similar early in the course of sepsis and are reasonable mimics of the onset of sepsis that occurs in patients. Very high doses of endotoxin resulted in sustained lung inflammation and AKI; AKI, but not lung inflammation, was sustained in the CLP model. These data suggest that a critical aspect of the use of animal models to study sepsis is recognition of the stage, severity, and timing of organ injury that is occurring in that model. It appears that 4 hours post sepsis in either endotoxin or CLP may represent established sepsis at presentation in patients. Our data highlight the need to study established disease and multiple injured organs in order to facilitate the translation of results in animal models to human clinical trials.
